# Circulating MicroRNA-21 and MicroRNA-122 as Prognostic Biomarkers in Hepatocellular Carcinoma Patients Treated with Transarterial Chemoembolization

**DOI:** 10.3390/biomedicines9080890

**Published:** 2021-07-25

**Authors:** Filippo Pelizzaro, Romilda Cardin, Anna Sartori, Angela Imondi, Barbara Penzo, Camillo Aliberti, Alberto Ponzoni, Alessandro Vitale, Umberto Cillo, Fabio Farinati

**Affiliations:** 1 Gastroenterology Unit, Department of Surgery, Oncology and Gastroenterology, University of Padova, 35128 Padova, Italy; filippo.pelizzaro@unipd.it (F.P.); romilda.cardin@unipd.it (R.C.); anna141290@gmail.com (A.S.); angelaimondi@gmail.com (A.I.); barbara.penzo@aopd.veneto.it (B.P.); 2 Radiology Unit, Azienda Ospedale-Università di Padova, 35128 Padova, Italy; camy.ali@libero.it (C.A.); alberto.ponzoni@aopd.veneto.it (A.P.); 3 Diagnostic Imaging Department, Pederzoli Hospital, Peschiera del Garda, 37019 Verona, Italy; 4 Hepatobiliary Surgery and Liver Transplantation Unit, Department of Surgery, Oncology and Gastroenterology, University of Padova, 35128 Padova, Italy; alessandro.vitale@unipd.it (A.V.); cillo@unipd.it (U.C.)

**Keywords:** hepatocellular carcinoma, HIF-1α, microRNA, prognosis, drug-eluting beads transarterial chemoembolization

## Abstract

Background: MicroRNAs (miRNAs) have been proposed as biomarkers in hepatocellular carcinoma (HCC). We aim at evaluating miR-21 and miR-122 in HCC patients treated with drug-eluting beads transarterial chemoembolization (DEB-TACE) as prognostic biomarkers and investigating their correlation with hypoxia inducible factor-1α (HIF-1α) serum levels. Methods: In this retrospective study, 12 healthy subjects, 28 cirrhotics, and 54 HCC patients (tested before and four weeks after DEB-TACE) were included. Whole blood miR-21 and miR-122 levels were measured by quantitative real time (qRT)-PCR, while serum HIF-1α was assessed by an enzyme-linked immunosorbent assay (ELISA) test. Results: The highest level of miR-21 was found in cirrhotics, while HCC patients had the highest level of miR-122 (which was even higher in “viral” HCC, *p* = 0.006). miR-21 ratio (after/before DEB-TACE) and miR-122 below their respective cut-offs identified patients with longer progression-free survival (*p* = 0.0002 and *p* = 0.02, respectively). The combined assessment of alpha-fetoprotein and miR-21 ratio, both independent prognostic predictors, identified early progressors among patients with complete or partial radiological response. miR-21 levels positively correlated with HIF-1α before (*p* = 0.045) and after DEB-TACE (*p* = 0.035). Conclusions: miR-21 ratio and miR-122 are useful prognostic markers after DEB-TACE. miR-21 correlates with HIF-1α and probably has a role in modulating angiogenesis in HCC.

## 1. Introduction

Hepatocellular carcinoma (HCC) is one of the most relevant cause of cancer-related death worldwide [1]. Among all the biomarkers proposed for HCC, only alpha-fetoprotein (AFP) has a worldwide clinical application, despite not being completely satisfactory [2]. As a consequence, there is a continuous search for new reliable biomarkers for the management of HCC patients, in particular in the predictive and prognostic settings.

MicroRNAs (miRNAs) are small non-coding single-stranded RNAs (≈22 nucleotides long), extensively involved in the regulation of gene expression. In carcinogenesis, they act on major tumor-related genes, either as oncogenes or as onco-suppressors [3]. Data on their role as biomarkers in HCC have been produced especially by Oriental authors in non-Caucasian populations and, in particular, miR-21 and miR-122 seem to be very promising. miR-21 is an onco-miRNA, detectable at high levels in tissue [4,5,6] and serum [7,8,9] of HCC patients. High levels of miR-21 after liver resection are predictive of disease-progression [6] and poor prognosis [5,7] while, in patients treated with loco-regional therapies, its role as a prognostic predictor is less clear [10,11,12]. miR-122, on the other hand, the most abundant liver-specific miRNA [13], acts as a tumor-suppressor reducing cancer cell proliferation, promoting apoptosis, and modulating drug resistance, invasion and metastasis [14]. Despite its down-regulation in HCC cells, miR-122 levels have been reported to be elevated in the serum of HCC patients compared to healthy controls [15,16], while its potential role in predicting HCC prognosis is still debatable.

Neoangiogenesis is one of the most important molecular pathways involved in HCC progression. miR-21 proved to be a regulator of angiogenesis in prostate, lung, and colorectal cancers, modulating hypoxia inducible factor-1α (HIF-1α) and vascular endothelial growth factor (VEGF) [17,18,19]. miR-122, as recently demonstrated, targets HIF-1α in diet-induced steatohepatitis [20], with some data suggesting an interplay between the two molecules also in HCC [21]. Moreover, a very recent paper found a role of miR-122 in enhancing liver ischemia tolerance in a murine model of hepatic ischemia-reperfusion injury through its induction by HIF-1α [22].

In this study, we aimed at comparing the levels of circulating miR-21 and miR-122 in healthy subjects, cirrhotics, and HCC patients and at evaluating the role of these miRNAs as predictors of progression-free survival (PFS) in a group of Caucasian HCC patients treated with drug-eluting beads transarterial chemoembolization (DEB-TACE). Moreover, we assessed the correlation of miR-21 and miR-122 with the circulating transcription factor HIF-1α before and after the treatment, which is able to profoundly stimulate angiogenesis by the induction of tumor ischemia [23].

## 2. Materials and Methods

### 2.1. Patients

In this study, blood samples from 12 healthy volunteers, 28 cirrhotics, and 54 HCC patients consecutively collected between July 2019 and April 2020, were retrospectively evaluated. Each subject provided written informed consent to participate to the study, which was conducted in accordance to the Declaration of Helsinki and was approved by the Ethics Committee of the Padova University Hospital (protocol code 46093, 12 August 2016).

Blood samples from cirrhotics were obtained in the outpatient’s clinic of the Gastroenterology Unit of the Padova University Hospital from patients with chronic liver disease fulfilling the following criteria: International Normalized Ratio (INR) >1.2, White Blood Cell <4.4 × 10^9^/L, Platelets <150 × 10^9^/L (at least two out of three), and abdomen ultrasonography (US) showing findings compatible with cirrhosis. All cirrhotic patients were regularly surveilled for the development of HCC with US every six months, and the presence of HCC was ruled out with dynamic computed tomography (CT) or magnetic resonance imaging (MRI) at the time of study entry.

HCC patients included in the study, diagnosed according to guidelines [24,25], were admitted to Gastroenterology Unit of Padova University Hospital for treatment (DEB-TACE). In all patients, chemoembolization was done using doxorubicin-loaded drug-eluting beads after super selective catheterization of the tumor feeding artery. In this subgroup, two blood samples were collected: the first immediately before DEB-TACE (t_0_) and the second four weeks after the procedure (t_1_), at the time of the control imaging performed in order to evaluate the efficacy of treatment.

The following clinical and tumor-related variables were recorded: sex, age, etiology, presence of clinically relevant portal hypertension (CRPH), main serological parameters (total bilirubin, INR, creatinine, albumin and AFP, the latter both at t_0_ and at t_1_ in patients with HCC), Child-Pugh class, Model for End-Stage Liver Disease (MELD) score and Eastern Cooperative Oncology Group performance status (ECOG-PS). CRPH was defined as presence of splenomegaly, esophageal varices or ascites, and platelets count <100 × 10^9^/L [26]. In HCC patients, number and size of liver nodules, presence of macrovascular invasion (MVI) and/or extrahepatic spread (EHS), evaluated before DEB-TACE with dynamic CT or MRI, were recorded. Patients were staged according to the Barcelona Clinic Liver Cancer (BCLC) system. The efficacy of DEB-TACE was evaluated with dynamic CT or MRI performed four weeks after the treatment and the Modified Response Evaluation Criteria In Solid Tumors (mRECIST) [27] were used to classify the radiological response in complete (CR), partial (PR), stable disease (SD), or progressive disease (PD).

### 2.2. RNA Isolation and MiRNAs Analysis

Ten milliliters of venous blood were collected from each patient: 5 mL of whole blood were used for RNA extraction, and the other 5 mL for serum and plasma separation. Samples were preserved at −80 °C till the assays.

Whole blood samples were used for the determination of miRNAs. Total RNA was extracted from 200 μL of whole blood using the Quick-RNA^TM^ Whole Blood extraction kit (Zymo Research, Irvine, CA, USA). Extraction efficiency was checked through adding synthetic oligonucleotides (UniSp2, UniSp4, UniSp5) at recommended concentrations. Reverse transcription for cDNA synthesis was performed using the miRCURY LNA RT kit (Qiagen, GmbH, Hilden, Germany) according to the manufacturer’s instructions. Reverse transcription efficiency was checked through adding synthetic oligonucleotides (UniSp6). The expression of miRNAs was evaluated by quantitative real time (qRT)-PCR analysis (miRCURY LNA miRNA PCR Assays and PCR Panels, Qiagen, GmbH, Hilden, Germany), according to the manufacturer’s instructions, on a PRISM 7900HT system (Applied Biosystems, Foster City, CA, USA) with miR-93, miR-103a, miR-425 as internal reference controls for normalization (levels of these control miRNAs are shown in Table S1). Each miRNA assay was replicated twice. The relative expression of each miRNA was calculated using the 2^−∆∆Ct^ (fold-change [fc]) method, using healthy subjects as the reference group for the normalization.

### 2.3. HIF-1α Assay

A commercial ELISA kit (Cloud-Clone Corp., Katy, TX, USA) was used to determine HIF-1α in the serum samples from cirrhotics and HCC, according to the manufacturer’s instructions. The amount of HIF-1α (ng/mL) was derived by interpolation of samples absorbance on the calibration curves plotted with calibrators. Briefly, plates precoated with an antibody specific to HIF-1α were incubated with 100 μL of serum. HIF-1α was revealed by the addition of Detection Reagent A and B at 450 nm.

### 2.4. Statistical Analysis

Quantitative variables were reported as median and interquartile range (IQR), while categorical variables as absolute frequency and percentage. Mann–Whitney and Wilcoxon matched-pairs signed rank tests were used to compare quantitative variables. The comparison between categorical data were performed with χ^2^ or Fischer’s exact tests. The correlations between continuous variables were established calculating the non-parametric Spearman coefficient.

PFS was calculated from the date of DEB-TACE to tumor progression or death, with data censored on 1 February 2021, and it was expressed as median and IQR. In the definition of the prognostic role of miRNAs, not only their values measured before DEB-TACE (t_0_), but also miR-21 and miR-122 ratios, defined as the ratio between t_1_ and t_0_ levels, were considered as potential biomarkers. The prognostic cut-offs of the markers (miR-21, miR-122 and their ratios; HIF-1α; AFP) were established using the receiver operating characteristic (ROC) curve method, taking as threshold the value with maximal sensitivity and specificity (Youden J test). The Kaplan–Meier method and the log-rank test were used to estimate and compare survival curves. The independent predictors of prognosis were assessed with the Cox multivariate regression analysis, including in the model only the variables significantly or borderline (*p* ≤ 0.10) associated with PFS in the univariate test. A *p*-value (two-tails) <0.05 was considered as significant in this study. IBM SPSS Statistics (Version 25.0, IBM Corp. Armonk, NY, USA) and GraphPad Prism (version 8.3.1, GraphPad Software, La Jolla, CA, USA) were used for all the calculations in this study.

## 3. Results

### 3.1. Baseline Characteristics

Baseline characteristics of cirrhotics and HCC patients included in the study are shown in Table 1. Cirrhotics and HCC patients were predominantly males, with similar age. Cirrhotics had mostly an alcohol-related liver disease, while HCV was the most frequent etiology in HCC patients (*p* = 0.07). Compared to HCC group, cirrhotics had more frequently CRPH (92.9% vs. 60.4%; *p* = 0.002) and a worse residual liver function (Child-Pugh A in 46.4% vs. 87.0%, *p* = 0.0002; and median MELD of 13 [IQR, 10–19] vs. 8 [7,8,9,10,11], *p* < 0.0001).

In HCC patients, the median number of liver nodules was 2 [1,2,3,4] with a median size of 2.2 cm [1.8–3.6]. The majority of patients were classified in BCLC stages A (46.3%) and B (37.0%), and 79.6% of patients had been previously treated, mostly with a combination of curative and intra-arterial therapies. The disease control rate after DEB-TACE was 81.5% (CR in 44.5%, PR in 29.6% and SD in 7.4% of patients).

### 3.2. Levels of Circulating MiRNAs

**Figure 1:**
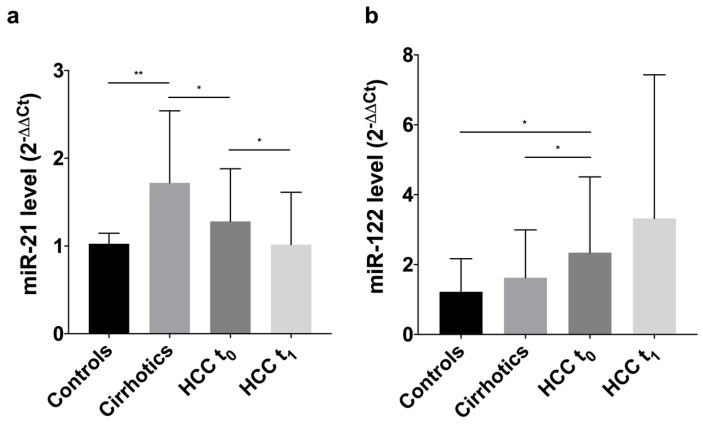
Histograms showing circulating levels of miR-21 and miR-122 in controls, cirrhotics, HCC patients at t_0_ and t_1_ (representing median with error bar showing the third quartile). (**a**) The median of miR-21 circulating levels is 1.03 fc [0.74–1.15] in controls, 1.72 fc [1.13–2.54] in cirrhotics, and 1.28 fc [0.78–1.88] in patients with HCC at t_0_. There is a significant difference in the circulating level between controls and cirrhotics (*p* = 0.009) and between cirrhotics and HCC patients (*p* = 0.047). In HCC, the miR-21 levels at t_0_ are significantly higher than those measured at t_1_ (1.02 fc [0.69–1.66]; *p* = 0.03); (**b**) the median of miR-122 circulating levels is 1.22 fc [0.39–2.17] in controls, 1.63 fc [0.51–2.99] in cirrhotics, and 2.34 fc [1.36–4.51] in HCC patients at t_0_. There is a statistically significant difference in the levels of the miRNA comparing HCC patients with controls (*p* = 0.02) and cirrhotics (*p* = 0.04). In HCC patients, no significant differences are present in t_0_ and t_1_ levels of miR-122. * *p* < 0.05; ** *p* ≤ 0.01.

Cirrhotic patients had a median level of miR-21 of 1.72 fc [1.13–2.54], significantly higher compared to healthy volunteers (1.03 fc [0.74–1.15]; *p* = 0.009) and HCC patients (1.28 fc [0.78–1.88]; *p* = 0.047). In HCC, a statistically significant drop of miR-21 after DEB-TACE was observed (1.02 fc [0.69–1.66]; *p* = 0.03), returning to levels comparable to those of healthy individuals (*p* = 0.76) (Figure 1a).

miR-122 showed a progressive increase from 1.22 fc [0.39–2.17] in controls to 1.63 fc [0.51–2.99] in cirrhotics and 2.34 fc [1.36–4.51] in HCC patients. It was significantly higher in HCC patients compared to controls (*p* = 0.02) and cirrhotics (*p* = 0.04). After TACE a further increase in miR-122 was observed, despite not statistically significant (3.41 fc [1.25–7.72]; *p* = 0.48) (Figure 1b).

**Figure 2:**
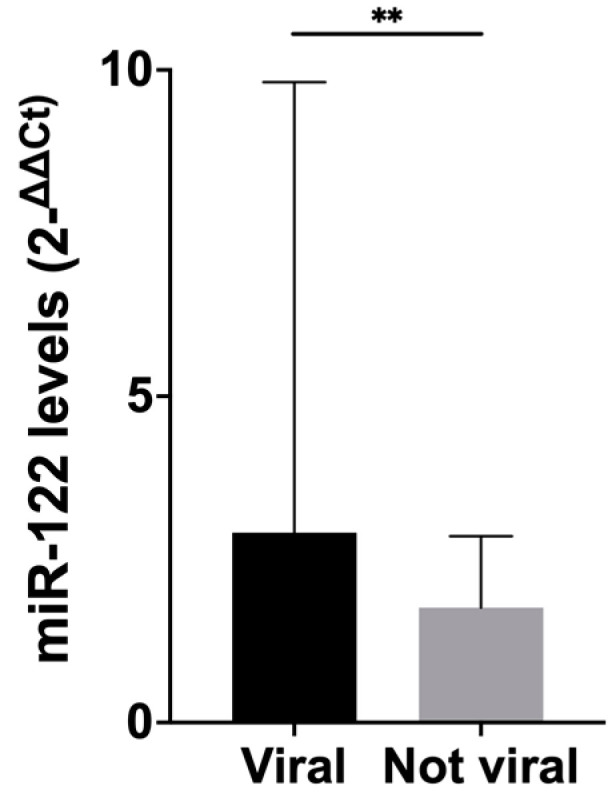
Histograms showing circulating levels of miR-122 according to the etiology of the underlying liver disease (representing median with error bar showing the third quartile). Patients with viral HCC had statistically significantly higher levels of circulating miR-122 compared to patients with alternative etiologies (*p* = 0.006). The median of miR-122 circulating levels in patients with viral etiology is 2.91 fc [1.62–9.82], a value statistically significant higher compared to the level registered in patients with alternative etiologies (1.76 fc [0.86–2.86]; *p* = 0.006). ** *p* ≤ 0.01.

In HCC patients, no association between circulating levels of miR-21 and any of the characteristics evaluated (sex, age, etiology, Child-Pugh class, MELD, tumor burden, BCLC stage) was observed, while miR-122 levels were associated only with etiology, being significantly higher in patients with a virus-related liver disease (HCV or HBV) compared to patients with alternative etiologies (2.91 fc [1.62–9.82] and 1.76 fc [0.86–2.86], respectively; *p* = 0.006) (Figure 2). No differences in miR-122 levels were found between HBV and HCV patients (2.82 fc [1.74–11.2] vs. 2.79 fc [1.37–9.77], respectively; *p* = 0.64).

AFP in cirrhotics (3.2 ng/mL [2.3–6.95]) was significantly lower than in HCC at t_0_ (6.85 ng/mL [3.05–17.23]; *p* = 0.009) and at t_1_ (6.1 ng/mL [3.2–26.7]; *p* = 0.003).

### 3.3. Survival Analysis

HCC patients had a median follow-up of 11.8 months [7.3–16.7] and all except 7 patients were alive at the end of the study. The median PFS was 3.9 months [1.4–8.3].

**Figure 3:**
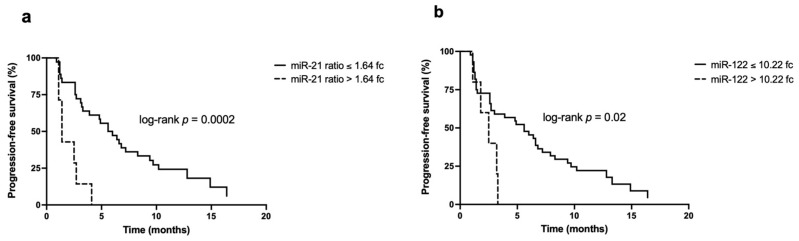
Kaplan–Meier curves for the PFS according to miR-21 ratio and miR-122 levels: (**a**) Patients with miR-21 ratio ≤1.64 fc have a significantly better PFS compared to patients with miR-21 ratio >1.64 fc (5.6 months [1.2–10.2] vs. 1.4 months [0.2–2.7]; *p* = 0.0002); (**b**) patients with miR-122 ≤10.22 fc have a statistically significant higher PFS compared to patients with miR-122 levels >10.22 fc (5.6 months [1.4–9.7] and 2.5 months [0.7–3.2]; *p* = 0.02).

The ROC curves used to identify the cut-off for miR-21, miR-21 ratio, miR-122, and miR-122 ratio are showed in Figure S1. miR-21 quantified before DEB-TACE, at the threshold identified with the ROC curve method (0.73 fc), was not able to discriminate patients according to their PFS (*p* = 0.17). However, patients with miR-21 ratio below its cut-off (1.64 fc) had a statistically significantly longer PFS compared to those with levels above 1.64 fc (median PFS 5.6 months [1.2–10.2] vs 1.4 months [1.1–2.7]; *p* = 0.0002) (Figure 3a).

Unlike miR-21, miR-122 levels measured at t_0_ were predictive of PFS: patients with miR-122 below the cut-off (10.22 fc) had a median PFS of 5.6 months [1.4–9.7], significantly longer than the 2.5 months [1.8–3.2] obtained in the comparator group (*p* = 0.02) (Figure 3b). No statistically significant differences in PFS were demonstrated with respect to miR-122 ratio at the cut-off of 0.87 fc, despite the longer PFS in patients with levels of the marker below the cut-off (5.8 months vs. 3.3 months, respectively; *p* = 0.9).

At the cut-off established with the ROC curve method and the Youden J test (7.5 ng/mL, Figure S2), AFP levels at t_0_ proved to be predictive of PFS, which was 6.6 months [2.7–13.3] in patients with values ≤7.5 ng/mL and 2.6 months [1.2–4.8] in the other group (*p* = 0.01). By contrast, HIF-1α at t_0_ (cut-off of 0.53 ng/mL, Figure S3) was not useful in predicting PFS (*p* = 0.26).

### 3.4. Univariate and Multivariate Analysis

miR-21 ratio, miR-122, AFP, radiological response, number of nodules, tumor size, presence of CRPH, and BCLC stage were associated with PFS at the univariate analysis. In the multivariate model, AFP (hazard ratio [HR] 4.31, 95% CI 1.66–11.20), miR-21 ratio (HR 8.61, 95% CI 2.03–36.47), and radiological response (HR 10.44, 95% CI 2.74–39.79) were singled out as independent predictors of PFS (Table 2).

**Figure 4:**
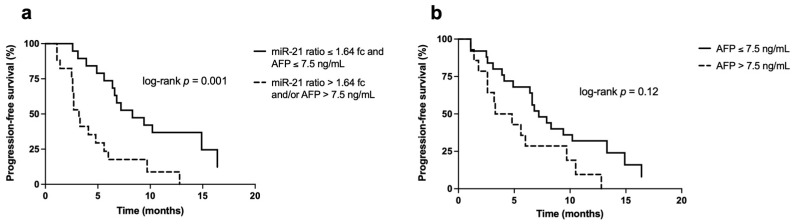
Kaplan–Meier curves for the PFS in patients with favorable radiological response (CR and PR). (**a**) Survival curves according to the combined evaluation of miR-21 ratio and AFP levels. Patients with both markers below their respective cut-offs achieved a statistically significant longer PFS compared to patients with at least one marker above its prognostic cut-off (8.3 months [6.4–16.4] in patients with miR-21 ratio ≤1.64 fc and AFP ≤7.5 ng/mL vs. 3.3 months [2.5–6.0] in the comparator group; *p* = 0.001); (**b**) survival curves of AFP alone in patients with CR and PR. Despite demonstrating a longer median PFS, patients with AFP ≤7.5 ng/mL had not a statistically significant higher survival compared to patients with AFP <7.5 ng/mL (7.2 months [4.1–13.3] vs. 4.1 months [2.6–9.7], respectively; *p* = 0.12).

Considering these results, we also evaluated whether the combined evaluation of miR-21 ratio and AFP was able to sub-stratify patients with a “favorable” radiological response (CR and PR) according to their PFS. We found a statistically significantly longer PFS in patients with both markers below their respective cut-offs (miR-21 ratio ≤1.64 fc and AFP ≤7.5 ng/mL) compared to those with at least one marker positive (8.3 months [6.4–16.4] and 3.3 months [2.5–6.0], respectively; *p* = 0.001) (Figure 4a). In the subset of patients with CR and PR, the determination of both biomarkers provided an advantage compared to the use of AFP alone, considering that patients with AFP ≤7.5 ng/mL had a longer but not statistically significant different PFS compared to those with AFP above the cut-off (median PFS of 7.2 months [4.1–13.3] vs. 4.1 months [2.6–9.7], respectively; *p* = 0.12) (Figure 4b).

### 3.5. Correlation between MicroRNAs and HIF-1α

**Figure 5:**
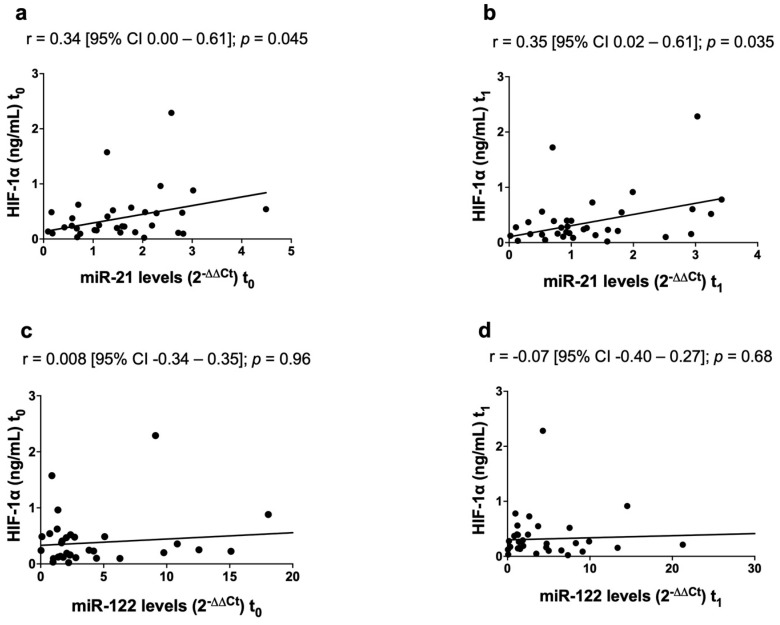
Correlations between the circulating levels of miR-21, miR-122 and HIF-1α in HCC patients. miR-21 was positively correlated with HIF-1α before (**a**) and after DEB-TACE (**b**). No statistically significant correlations were found between miR-122 and HIF-1α, both before (**c**) and after DEB-TACE (**d**).

HIF-1α levels were significantly higher in cirrhotics (0.43 ng/mL [0.32–0.54]) than in HCC patients, both before (0.23 ng/mL [0.12–0.49]; *p* = 0.02) and after DEB-TACE (0.23 ng/mL [0.12–0.46]; *p* = 0.009). In HCC patients miR-21, but not miR-122, was positively correlated with HIF-1α both at t_0_ (r = 0.34 [95% CI 0.00–0.61]; *p* = 0.045) and at t_1_ (r = 0.35 [95% CI 0.02–0.61]; *p* = 0.035) (Figure 5). In cirrhotics no correlations were found between miRNAs and HIF-1α.

## 4. Discussion

The recently updated European guidelines on HCC management identified as an unmet need the development of useful prognostic and predictive biomarkers [25]. Several studies evaluated miR-21 and miR-122 role in promoting HCC development and progression [14,28,29,30,31,32,33], and, despite the amount of publications about their potential role as biomarkers, data are not conclusive. In particular, there are conflicting results regarding their prognostic role and the precise clinical and therapeutic setting in which they could be useful is not completely clear [34,35]. The majority of data on these miRNAs as biomarkers in HCC come from studies on eastern populations, quite different from the western ones in terms of etiology and severity of liver disease [36]. With this in mind, we assessed the prognostic efficiency of miR-21 and miR-122 in a group of Caucasian HCC patients treated with DEB-TACE. Unlike the majority of reports published, in this study circulating miRNAs were measured in whole blood samples rather than in serum or in plasma. The rationale behind this choice is that recently, in pancreatic, ovarian, lung, and gallbladder cancers, miRNAs evaluated in whole blood samples proved to be more accurate [37]. Among the advantages on using whole blood samples, there are a higher miRNA yield and fewer errors than when using serum or plasma samples [37].

In our cohort, miR-21 levels were higher in cirrhotics than in controls and in HCC patients, without differences between the latter two. In HCC, miR-21 levels are reported to be higher in comparison to heathy subjects [9,38], but things become less clear when HCC and chronic liver disease are compared. Guo et al. [38] reported higher levels of miR-21 in HCC patients with respect to both chronic hepatitis B and liver cirrhosis patients; in contrast Pu et al. [9] and Xu et al. [15] concluded that miR-21 expression was higher in chronic hepatitis B patients than in HCC patients. In our study, as already reported after TACE [11], miR-21 levels showed a statistically significant decline, returning to levels comparable to those found in controls, confirming its pro-oncogenic role.

We detected higher levels of miR-122 in HCC patients compared to healthy controls, in line with what is already known [15,39], and compared with cirrhotics. On the latter point the published studies are again not concordant: besides studies reporting higher miR-122 in HBV-infected patients compared to HCC [15,39], others claimed no significant differences [16,40] or even higher levels in HCC patients [8]. Considering its role as tumor-suppressor and its down-regulation in HCC tissue compared with adjacent benign liver [14,28,33], the finding of higher circulating miR-122 in HCC patients is not easy to explain. miR-122 levels might reflect liver injury more than the presence of the tumor itself. Indeed, some studies correlated hepatic inflammation and cell death in patients with HBV and HCV chronic hepatitis with serum miR-122 levels [41,42]. The mild increase of miR-122 after DEB-TACE found in our study might be explained speculating that its levels at t_1_ do not reflect the effectiveness of treatment, but instead the concomitant liver injury. The lack of a statistical significance in this difference is probably due to the long temporal interval between the treatment and the evaluation of miRNA in our experimental setting, with a shorter time more likely resulting in larger differences. Nevertheless, it should be underlined that lower levels of miR-122 seven days after TACE have been reported, in contrast with our results [11].

miR-122 levels were higher in patients with HCC developed on a virus-related liver disease. This was not an unexpected finding, considering that miR-122 is involved in HBV genes expression [43] and it has a role in stimulating HCV replication [44]. This association with HBV/HCV etiology was not confirmed in cirrhotic patients, probably because of the small number of patients in this group, who had an alcohol-related liver disease in the majority of cases.

The prognostic role of miR-21 has been extensively studied after surgery [5,6,7,8], but in patients undergoing loco-regional treatments data are not conclusive. High plasma miR-21 levels were not found to be associated with survival after TACE from some authors [10,11], while others found an association only at univariate analysis [12]. Here, we confirmed that miR-21 is not a predictor of PFS when evaluated at t_0_, but when the marker is considered as the ratio before and after treatment, in a dynamic way, it could be predictive of PFS, with Kaplan–Meier curves showing an impressive divergence.

The accuracy of miR-122 as a prognostic marker has not been clearly established. In patients treated with liver resection high serum levels of miR-122 appeared to correlate with longer survival [45], while the opposite was true for patients treated with radiofrequency ablation [46]. In other authors’ experience, miR-122 had no prognostic role in general [8] or, specifically, in TACE-treated patients [47]. In our study, miR-122 was useful in predicting prognosis when evaluated at t_0_, as the patients with lower levels had a significantly longer PFS (again with an important divergence of Kaplan–Meier curves). Conversely, no significant association with PFS was demonstrated for miR-122 ratio, despite the fact that patients with lower values showed a slightly, not statistically significant, longer median survival.

For both miR-21 and miR-122, our results are in contrast to those published by *Suehiro* et al. [11], who identified only miR-122 ratio as a prognostic marker (longer survival in patients with higher ratio levels). Despite similar experimental designs, the two studies are not completely comparable: in the *Suehiro* et al. study the second sample was obtained 7 days after TACE, miRNAs levels were measured in extracellular vesicles, patients were treated with conventional and not DEB-TACE, and different internal reference controls were used for normalization.

At the Cox multivariate analysis, radiologic response, miR-21 ratio, and AFP levels were identified as independent predictors of PFS, in this order in terms of HR. In other words, miR-21 ratio had a higher impact than AFP levels. We also wondered whether assessing miR-21 ratio and AFP in combination could be useful to stratify patients with CR/PR according to their PFS and we found that the subgroup of patients with both miR-21 ratio and AFP below their respective cut-offs had significantly longer PFS than patients with at least one marker above the cut-off. Moreover, in this sub-group of patients with favorable radiological response, the combined determination of AFP and miR-21 ratio provided a better stratification according to PFS compared to AFP alone. In fact, we found no statistically significant difference between patients with AFP below and above the cut-off of 7.5 ng/mL. These results strengthen the role of miR-21 ratio in identifying the subgroup of patients with early progressing HCC.

Among the several molecular pathways in which they are involved, miRNAs play a role in modulating neoangiogenesis in human cancers. In prostate, lung, and colorectal cancers, miR-21 regulates the expression of HIF-1α and VEGF [17,18,19], but this association was not confirmed in human HCC tissue [4]. Moreover, HIF-1α proved to be a miR-122 target in diet-induced steatohepatitis [20] and in a mouse model of HCC [21]. A very recent paper provided more insights about the interplay between miR-122 and hypoxia-induced pathways in a murine model of ischemia-reperfusion injury [22]. With this in mind, we evaluated the correlation between miR-21, miR-122, and HIF-1α in our group of patients treated with DEB-TACE, a treatment that induces liver ischemia and, in turn, over-expression of hypoxia-related genes. A positive mild correlation between miR-21 and HIF-1α in HCC patients both before and after the treatment was found, while no significant associations were found with miR-122. To the best of our knowledge, our study is the first to link miR-21 with HIF-1α in human HCC treated with DEB-TACE. The correlation found does not necessarily imply the causality of the relationship, but our results are consistent with the hypothesis of a miR-21 involvement in regulating the angiogenic pathway also in HCC, as already demonstrated for other malignancies [17,18,19], and paves the way for additional studies aimed at demonstrating this assumption. 

Among the limitations of our study, the most important one is its retrospective nature that might have introduced unintentional biases. However, these biases are mitigated by the fact that patients were consecutively collected. The relatively small sample size (especially looking to cirrhotic patients), and the baseline differences between HCC and cirrhotics (particularly in residual liver function), could have prevented us to reach more definite conclusions in the comparison of miRNAs levels between groups.

There are many differences between our results and those reported in other publications. The comparison of different studies on miRNAs is difficult because a homogeneous methodological protocol in miRNA evaluation has not yet been reached. In particular, the differences between studies are related to the endless list of confounders that includes samples size and selection, type of biologic samples used for the miRNAs assay (plasma, serum, exosomes, whole blood), RNA extraction procedures, internal controls, control groups for data normalization, methodology to express miRNA levels. Moreover, the majority of data in literature derives from eastern countries in which the leading cause of HCC and chronic liver disease is HBV infection, and with a larger share of tumors developing on a non-cirrhotic background [36]. In western countries, by contrast, the vast majority of HCC patients had an underlying cirrhosis, the most frequent etiologies being HCV infection and alcohol.

We selected PFS as end-point because overall survival, the ideal end-point in oncology, was not evaluable considering the very short follow-up of patients enrolled. It must be kept in mind however that PFS could be considered a suitable surrogate of overall survival, particularly in diseases in which multiple lines of active treatment are available, such as HCC [48].

## 5. Conclusions

In conclusion, according to our results, miR-21 ratio and miR-122 predict PFS after TACE and propose themselves as useful prognostic markers. In particular, miR-21 ratio is associated to PFS at univariate and multivariate analysis and identifies (together with AFP) early progressors among the patients achieving a radiological response to treatment. In addition, a correlation between circulating HIF-1α and miR-21 expression was found, suggesting a possible role of the latter in modulating angiogenesis also in HCC, as it does in other type of tumors. Additional studies, possibly prospective, and the development of widely shared methodological protocols are necessary before the introduction of miRNAs quantification in clinical practice, but our results look quite promising.

## Figures and Tables

**Table 1:** Baseline characteristics of cirrhotic and HCC patients.

Variable		Cirrhotics (*n* = 28)	HCC (*n* = 54)	*p* ^†^
Males—*n* (%)		20 (71.4)	44 (81.5)	0.40
Age (years)		63.5 (49.3–72.0)	67.0 (61.8–76.0)	0.13
Cirrhosis—*n* (%)		28 (100)	48 (88.9)	0.09
Etiology—*n* (%)	HBV	3 (10.7)	6 (11.1)	0.07
	HCV	4 (14.3)	20 (37.1)	
	Alcohol	16 (57.1)	16 (29.6)	
	Other	5 (17.9)	12 (22.2)	
CRPH—*n* (%)		26 (92.9)	33 (60.4)	0.002
Child-Pugh A—*n* (%)		13 (46.4)	47 (87.0)	0.0002
MELD score		13 (10–19)	8 (7–11)	<0.0001
Bilirubin (μmol/L)		23.5 (13.4–68.6)	15.0 (10.0–20.0)	0.006
Albumin (g/dL)		3.5 (2.9–4.0)	4.0 (3.5–4.3)	0.007
INR		1.32 (1.15–1.60)	1.12 (1.09–1.21)	0.0003
Number of nodules			2 (1–4)	
Diameter (cm)			2.2 (1.8–3.6)	
MVI and/or EHS—*n* (%)			4 (7.5)	
BCLC stage—*n* (%)	0/A		30 (55.6)	
	B/C		24 (44.4)	
Previous treatments—*n* (%)	LR/ABL		13 (24.0)	
	TACE		9 (16.7)	
	ABL/LR + TACE		21 (38.9)	
	No		11 (20.4)	
Radiological response	CR		24 (44.5%)	
	PR		16 (29.6%)	
	SD		4 (7.4%)	
	PD		10 (18.5%)	

^†^ Mann–Whitney test, χ^2^ test and Fischer’s exact test, as appropriate. Continuous data are expressed as median (interquartile range), while categorical data are presented as absolute frequency (percentage). Abbreviations: HCC, hepatocellular carcinoma; CRPH, clinically relevant portal hypertension; MELD, Model of End Stage Liver Disease; INR, international normalized ratio; MVI, macrovascular invasion; EHS, extrahepatic spread; BCLC, Barcelona Clinic Liver Cancer; ABL, ablation; LR, liver resection; TACE, transarterial chemoembolization; CR, complete response; PR, partial response; SD, stable disease; PD, progressive disease.

**Table 2:** Univariate and multivariate Cox analysis for factors independently associated with PFS.

Variables		Univariate Analysis		Multivariate Analysis	
		HR (95% CI)	*p*	aHR (95% CI)	*p*
AFP (ng/mL)	≤7.5	Ref	-	Ref	-
	>7.5	2.52 (1.37–4.66)	0.003	4.31 (1.66–11.20)	0.003
miR-21 ratio (2^−^^ΔΔ^^Ct^)	≤1.64	Ref	-	Ref	-
	>1.64	4.95 (1.93–12.65)	0.001	8.61 (2.03–36.47)	0.003
miR-122 (2^−^^ΔΔ^^Ct^)	≤10.22	Ref	-	Ref	-
	>10.22	2.98 (1.10–8.09)	0.03	2.11 (0.46–9.78)	0.3
Radiological response	CR/PR	Ref	-	Ref	-
	SD/PD	6.37 (2.91–13.95)	<0.0001	10.44 (2.74–39.79)	0.001
Number of nodules	≤3	Ref	-	Ref	-
	>3	2.30 (1.24–4.25)	0.008	0.51 (0.12–2.16)	0.4
Diameter (cm)	≤5	Ref	-	Ref	-
	>5	2.23 (0.97–5.16)	0.06	1.78 (0.47–6.78)	0.4
CRPH	No	Ref	-	Ref	-
	Yes	1.71 (0.92–3.17)	0.09	0.56 (0.23–1.35)	0.2
BCLC stage	0/A	Ref	-	Ref	-
	B/C	2.54 (1.36–4.74)	0.003	3.50 (0.77–15.96)	0.1

Abbreviations: HR, hazard ratio; CI; confidence interval; aHR, adjusted hazard ratio; Ref, reference; AFP, alpha-fetoprotein; CR, complete response, PR, partial response; SD, stable disease, PD, progressive disease; CRPH, clinically relevant portal hypertension; BCLC, Barcelona Clinic Liver Cancer.

## Data Availability

Not applicable.

## Supplementary Materials

The following are available online at https://www.mdpi.com/article/10.3390/biomedicines9080890/s1. Table S1. Levels of miRNAs (miR-93, miR-103a, miR-425) used as internal reference controls for normalization in our study. Figure S1. ROC curves used to identify the cut-off for miR-21 (a), miR-21 ratio (b), miR-122 (c), and miR-122 ratio (d). Figure S2. ROC curve for the identification of AFP cut-off. Figure S3. ROC curve for the identification of HIF-1α cut-off.
