# Supplementary material for: Circulating MicroRNA-21 and MicroRNA-122 as Prognostic Biomarkers in Hepatocellular Carcinoma Patients Treated with Transarterial Chemoembolization
Source: Biomedicines. 2021 Jul 25;9(8):890. doi: 10.3390/biomedicines9080890 (PMC8389644; doi:10.3390/biomedicines9080890)
Supplement: Supplementary file 1 [file biomedicines-09-00890-s001.zip › biomedicines-1300829-supplementary.pdf]

Table S1. Levels of miRNAs (miR-93, miR-103a, miR-425) used as internal reference controls for normalization in our study.

	miR-93	miR-103a	miR-425
Healthy subjects	13.60 ± 0.56 ct	13.37 ± 0.62 ct	16.12 ± 0.77 ct
Cirrhotics	13.89 ± 1.41 ct	13.72 ± 1.47 ct	16.14 ± 1.32 ct
HCC pre-TACE	14.79 ± 1.91 ct	14.67 ± 2.02 ct	16.78 ± 1.52 ct
HCC post-TACE	15.11 ± 2.11 ct	15.06 ± 2.30 ct	17.12 ± 1.63 ct

miRNA levels, expressed as threshold cycles (ct), are reported in the table as mean ± standard deviation (SD).

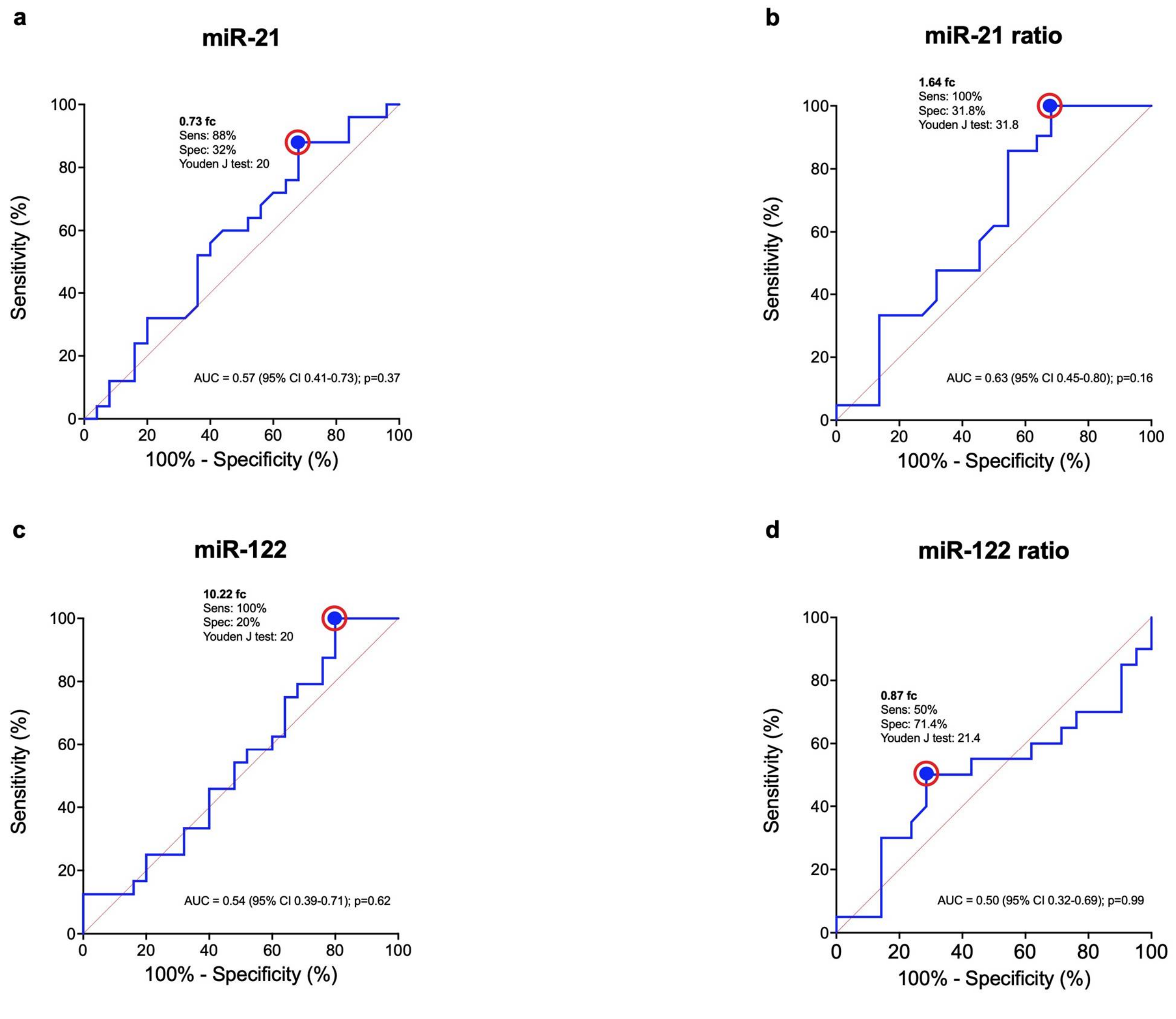

Figure S1. ROC curves used to identify the cut-off for miR-21 (**a**), miR-21 ratio (**b**), miR-122 (**c**) and miR-122 ratio (**d**).

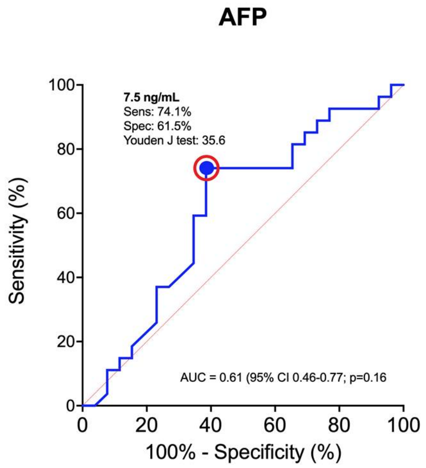

Figure S2. ROC curve for the identification of AFP cut-off.

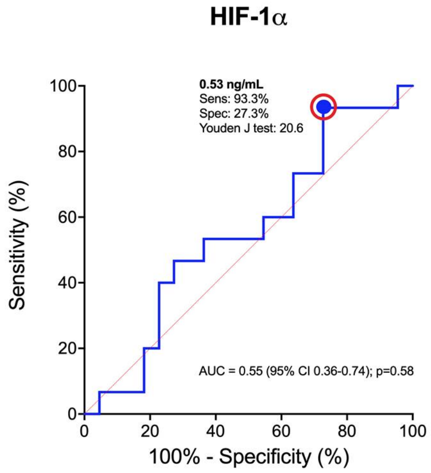

Figure S3. ROC curve for the identification of HIF-1α cut-off.
